# True Politeness

**Published:** 1866-07

**Authors:** 


### TRUE POLITENESS

Is benevolence personified, it is the practice of kindness. There is virtue even in the form of politeness ; it may be merely mechanical, still, like an air cushion, although there is nothing in it, it is very comfortable in use. Why not cultivate a pleasant mode of recognition for every one we meet on the street, however slight the acquaintance ? it would many a time lighten the load of some sorrowing heart, or cause some new resolve to " try again " when on the very verge of utter hopelessness, by the inspiration of the feeling " there's somebody at least cares a little for me." It elevates the lowly to have their superiors greet them courteously ; it unwittingly to themselves, begets a resolution to act more worthy of such recognition ; to earn it by a better behavior, a more tidy dress, a more dignified deportment.

